# Parameter Estimation and Model Selection in Computational Biology

**DOI:** 10.1371/journal.pcbi.1000696

**Published:** 2010-03-05

**Authors:** Gabriele Lillacci, Mustafa Khammash

**Affiliations:** Center for Control, Dynamical Systems and Computation, University of California at Santa Barbara, Santa Barbara, California, United States of America; California Institute of Technology, United States of America

## Abstract

A central challenge in computational modeling of biological systems is the determination of the model parameters. Typically, only a fraction of the parameters (such as kinetic rate constants) are experimentally measured, while the rest are often fitted. The fitting process is usually based on experimental time course measurements of observables, which are used to assign parameter values that minimize some measure of the error between these measurements and the corresponding model prediction. The measurements, which can come from immunoblotting assays, fluorescent markers, etc., tend to be very noisy and taken at a limited number of time points. In this work we present a new approach to the problem of parameter selection of biological models. We show how one can use a dynamic recursive estimator, known as extended Kalman filter, to arrive at estimates of the model parameters. The proposed method follows. First, we use a variation of the Kalman filter that is particularly well suited to biological applications to obtain a first guess for the unknown parameters. Secondly, we employ an a posteriori identifiability test to check the reliability of the estimates. Finally, we solve an optimization problem to refine the first guess in case it should not be accurate enough. The final estimates are guaranteed to be statistically consistent with the measurements. Furthermore, we show how the same tools can be used to discriminate among alternate models of the same biological process. We demonstrate these ideas by applying our methods to two examples, namely a model of the heat shock response in *E. coli*, and a model of a synthetic gene regulation system. The methods presented are quite general and may be applied to a wide class of biological systems where noisy measurements are used for parameter estimation or model selection.

## Author Summary

Parameter estimation is a key issue in systems biology, as it represents the crucial step to obtaining predictions from computational models of biological systems. This issue is usually addressed by “fitting” the model simulations to the observed experimental data. Such approach does not take the measurement noise into full consideration. We introduce a new method built on the combination of Kalman filtering, statistical tests, and optimization techniques. The filter is well-known in control and estimation theory and has found application in a wide range of fields, such as inertial guidance systems, weather forecasting, and economics. We show how the statistics of the measurement noise can be optimally exploited and directly incorporated into the design of the estimation algorithm in order to achieve more accurate results, and to validate/invalidate the computed estimates. We also show that a significant advantage of our estimator is that it offers a powerful tool for model selection, allowing rejection or acceptance of competing models based on the available noisy measurements. These results are of immediate practical application in computational biology, and while we demonstrate their use for two specific examples, they can in fact be used to study a wide class of biological systems.

## Introduction

Many biological processes are modeled using ordinary differential equations (ODEs) that describe the evolution over time of certain quantities of interest. At the molecular level, the variables considered in the models often represent concentrations (or number of molecules) of chemical species, such as proteins and mRNA. Once the pathway structure is known, the corresponding equations are relatively easy to write down using widely accepted kinetic laws, such as the law of mass action or the Michaelis-Menten law.

In general the equations will depend on several parameters. Some of them, such as reaction rates, and production and decay coefficients have a physical meaning. Others might come from approximations or reductions that are justified by the structure of the system and, therefore, they might have no direct biological or biochemical interpretation. In both cases, most of the parameters are unknown. While sometimes it is feasible to measure them experimentally (especially those in the first class), in many cases this is very hard, expensive, time consuming, or even impossible. However, it is usually possible to measure some of the other variables involved in the models (such as abundance of chemical species) using PCR, immunoblotting assays, fluorescent markers, and the like.

For these reasons, the problem of *parameter estimation*, that is the indirect determination of the unknown parameters from measurements of other quantities, is a key issue in computational and systems biology. The knowledge of the parameter values is crucial whenever one wants to obtain quantitative, or even qualitative information from the models [1],[2].

In the last fifteen years a lot of attention has been given to this problem in the systems biology community. Much research has been conducted on the applications to computational biology models of several optimization techniques, such as linear and nonlinear least-squares fitting [3], simulated annealing [4], genetic algorithms [5], and evolutionary computation [6],[7]. The latter is suggested as the method of choice for large parameter estimation problems [7]. Starting with a suitable initial guess, optimization methods search more or less exhaustively the parameter space in the attempt to minimize a certain cost function. This is usually defined as the error in some sense between the output of the model and the data that comes from the experiments. The result is the set of parameters that produce the *best fit* between simulations and experimental data. One of the main problems associated with optimization methods is that they tend to be computationally expensive and may not perform well if the noise in the measurements is significant.

Considerable interested has also been raised by Bayesian methods [8], which can extract information from noisy or uncertain data. This includes both measurement noise and intrinsic noise, which is well known to play an important role in chemical kinetics when species are present in low copy numbers [9]. The main advantage of these methods is their ability to infer the whole probability distributions of the parameters, rather than just a point estimate. Also, they can handle estimation of stochastic systems with no substantial modification to the algorithms [10]. The main obstacle to their application is computational, since analytical approaches are not feasible for non-trivial problems and numerical solutions are also challenging due to the need to solve high-dimensional integration problems. Nonetheless, the most recent advancements in Bayesian computation, such as Markov chain Monte Carlo techniques [11], ensemble methods [12],[13], and sequential Monte Carlo methods that don't require likelihoods [10],[14] have been successfully applied to biological systems, usually in the case of lower-dimensional problems and/or availability of a relatively high number of data samples. Maximum-likelihood estimation [15],[16] has also been extensively applied.

More recently, parameter estimation for computational biology models has been tackled in the framework of control theory by using state observers. These algorithms were originally developed for the problem of state estimation, in which one seeks to estimate the time evolution of the unobserved components of the state of a dynamical system. The controls literature on this subject is vast, but in the context of biological or biochemical systems the classically used approaches include Luenberger-like [17], Kalman filter based, [18]–[20], and high-gain observers [21]. Other methods have been developed by exploiting the special structure of specific problems [22]. State observers can be employed for parameter estimation using the technique of state extension, in which parameters are transformed into states by suitably expanding the system under study [22]–[24]. In this context extended Kalman filtering [25],[26] and unscented Kalman filtering [27] methods have been applied as well.

When the number of unknown parameters is very large, it is often impossible to find a unique solution to this problem. In this case, one finds several sets of parameters, or ranges of values, that are all equally likely to give a good fit. This situation is usually referred to as the model being *non identifiable*, and it is the one that's most commonly encountered in practice. Furthermore, it is known that a large class of systems biology models display sensitivities to the parameter values that are roughly evenly distributed over many orders of magnitude. Such “sloppiness” has been suggested as a factor that makes parameter estimation difficult [28]. These and similar results indicate that the search for the exact individual values of the parameters is a hopeless task in most cases [6]. However, it is also known that even if the estimation process is not able to tightly constrain any of the parameter values, the models can still be able to yield significant quantitative predictions [12].

The purpose of the present contribution is to extend the results on parameter estimation by Kalman filtering by introducing a procedure that can be applied to large parameter spaces, can handle sparse and noisy data, and provides an evaluation of the statistical significance of the computed estimates. To achieve this goal, we introduce a constrained hybrid extended Kalman filtering algorithm, together with a measure of accuracy of the estimation process based on a 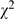 variance test. Furthermore, we show how these techniques together can be also used to address the problem of model selection, in which one has to pick the most plausible model for a given process among a list of candidates. A distinctive feature of this approach is the ability to use information about the statistics of the measurement noise in order to ensure that the estimated parameters are statistically consistent with the available experimental data.

The rest of this paper is organized as follows. In the Methods Section we introduce all the theory associated with our procedure, namely the constrained hybrid extended Kalman filter, the accuracy measure and its use in estimation refinement, and the application to the model selection problem. In the Results Section we demonstrate the procedure on two examples drawn from molecular biology. Finally, in the Discussion Section we summarize the new procedure, we give some additional remarks, and we point out how these findings will be of immediate interest to researchers in computational biology, who use experimental data to construct dynamical models of biological phenomena.

## Methods

### Problem formulation

Throughout this paper, we will assume that the process of interest can be modeled by a system of ordinary differential equations of the form:
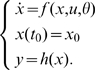
(1)The state vector 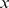 usually contains concentrations of certain chemical species of interest, such as mRNA or proteins. The *input signal* 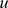 represents some kind of external forcing of the process, such as temperature changes, the addition or removal of certain chemicals or drugs, and so forth. The *output signal* 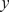 represents the quantity or quantities we can measure experimentally. These are related to the state 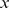 through the function 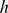, which we call the *output function*. The output function is to be determined from the design of the biological experiments that are used to get the measurements for parameter estimation. As an example, when measuring protein concentrations, in some biological experiments it is harder and/or more expensive and/or more time consuming to distinguish among different post-translational modifications of the same protein. This situation corresponds in our setting to choosing 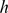 equal to the sum of two or more state variables, representing the total amount of protein.

The vector 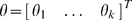 contains the unknown parameters that we seek to estimate. Note that, since the parameters are constants, it is always possible to consider them as additional state variables with a rate of change equal to zero. In this way, we treat them as constant *functions of time* as opposed to constant *numbers*. This technique is usually referred to as *state extension*. Our system (1) then becomes:
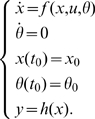
(2)


Using state extension, the problem of parameter estimation is converted into a problem of *state estimation*, that is determining the state of a system from measurements of the output. More precisely, we are trying to determine the *initial conditions* that when used to initialize the system (2) generate the observed output 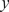. In the case of the parameters, since 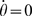, it is obvious that 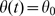 for all 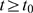.

Solving this problem requires answering the following two questions.

1. Given a system of the form (2), does the output 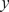 contain enough information to uniquely determine a reliable estimate of 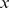 and 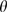
?
2. If so, how can we compute such estimate?

The first question is usually referred to as the problem of *identifiability*. In control theory, much work has been done in studying this property in terms of another one called *observability* [23],[24]. Roughly speaking, a system is observable if every set of initial conditions produces an output that is different from the one generated by every other set. Identifiability can also be studied a posteriori [6], by testing the reliability of the estimates *after they have been computed*. We will make use of this second approach.

To answer the second question, we need to show how to design an algorithm (or device) that can estimate 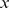 and 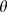 from measurements of 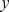, which, in general, will not be perfect, but noisy and sparse. Such algorithms, called *state observers*, can be formulated in a plethora of different ways, each of which is better suited for different applications. The observer we are going to use is based on extended Kalman filtering, and is described in detail in the next Section.

### Extended Kalman filtering

Extended Kalman filtering is considered to be the de-facto standard of nonlinear state estimation [29]. It found several applications in many different fields, such as positioning systems, robot navigation and economics. The Kalman filter is a set of equations that provides an efficient computational technique to estimating the state of a process, in a way that minimizes the covariance of the estimation error. The filter is very powerful in several aspects: it supports estimations of past, present, and even future states, and it can do so even when the precise nature of the modeled system is unknown. Unlike most of the classical parameter estimation methods, the Kalman filter is a *recursive estimator*. At each time step the filter refines the previous estimate by incorporating in it new information from the model and from the output.

The Kalman filter works in two steps: first it estimates the process state and covariance at some time using information from the model only (*prediction*); then it employs a feedback from the noisy measurements to improve the first estimates (*correction*). As such, the equations for the Kalman filter fall into two groups: *time update equations* for the prediction step and *measurement update equations* for the correction step. The time update equations are responsible for propagating forward (in time) the current state and error covariance estimates to obtain the *a priori* estimates for the next time step. The measurement update equations are responsible for the feedback, i.e. for incorporating a new measurement into the a priori estimate to obtain an improved *a posteriori estimate*. After each time and measurement update pair, the process is repeated with the previous a posteriori estimates used to predict the new a priori estimates.

In order to set these ideas in a more rigorous mathematical framework, consider the following system:
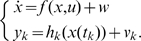
(3)As we note from the structure of system (3), we are assuming that we have a continuous-time process which we want to estimate using discrete-time measurements of the output. This is the most common case when dealing with deterministic models of biological systems. These are usually of the form (1), therefore continuous-time. However, the measurements for estimation tend to be available only at discrete time instants. We will denote these instants 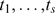, with 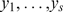 being the corresponding values of the measurements. The output of the filter will then be the a posteriori estimates of the state corresponding to instants 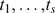, which we will denote 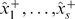. We remark that after applying state extension as described in the previous Section, the unknown parameters are now part of the state of the system, therefore their estimates at time 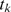 are components of 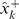. We also note that the output function 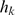 in (3) is allowed to be different at different time step: this is very important e.g. when incorporating data from different measurements, because it allows the algorithm to use measurements of different species at different times.

The variable 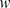, usually called the *process noise*, represents the amount of confidence we have in our model. The process noise is assumed to be a Gaussian random variable with zero mean and covariance 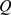, where 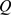 is a positive definite matrix. The noise that affects the different components of the state is assumed to be uncorrelated, so that 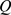 is diagonal. Larger entries in 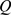 correspond to lower confidence in the accuracy of the model. The variable 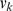 is referred to as the *measurement noise*, and expresses the reliability of the measurements. The measurement noise is also assumed to be Gaussian with zero mean, and its covariance matrix will be denoted by 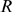. Again, 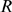 is assumed to be a positive definite, diagonal matrix, since the noise that affects different measurements is assumed to be uncorrelated. Note that while 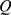 is usually chosen by the user in order to tell the filter how much the model should be trusted, 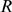 is fixed by the quality of the measurements. In other words, *the statistics of the measurements noise are assumed to be known*. This fact will be particularly important for the a posteriori reliability test described in the next Section.

The variation of the Kalman filter we present here is the one that is best suited for a system of the form (3), and it is usually referred to as the *hybrid extended Kalman filter* (HEKF). The word *extended* refers to the fact that it can deal with nonlinear systems, while *hybrid* indicates that it uses continuous-time process model and discrete-time measurements. We next describe the time update equations and measurement update equations of the HEKF.

First of all, we need some initial conditions to start the filter from. Ideally, we would like the initial conditions to be 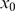 (the initial conditions of the process) but this is clearly not possible. Since we do not have any measurements available to estimate 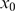, it makes sense to take our initial estimate of 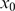 equal to the expected value of the initial state 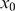. Therefore, we write:
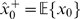
(4)It follows that the initial condition for the error covariance can be set as:
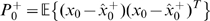
(5)


We can now apply the time update equations to obtain the current a priori estimates. The current a priori state estimate, which we denote 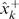, is formed by integrating the continuous-time process in the time interval 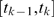, using the previous a posteriori estimate as initial condition. The current a priori error covariance estimate, denoted 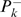, is formed by integrating a differential Lyapunov equation using the previous a posteriori error covariance as initial condition [29].
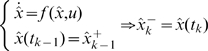
(6a)

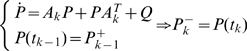
(6b)


The matrix 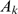 is the Jacobian of 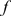 evaluated at the previous a posteriori state estimate. The structure of equations (6) shows a very important feature of the HEKF algorithm, i.e. its ability to deal with non-uniformly sampled data. As we will see in the examples in the Results Section, this is useful because it allows one to capture all the information about the evolution of a process using a minimum number of data points.

The measurement update equations are used to form the a posteriori estimates by incorporating information from the output of the system into the a priori estimates. The correction is based on the difference between the actual measurement and the *predicted measurement*, that is what the measurement would be if the real value of the state were exactly equal to its a priori estimate. Such difference is weighed by a *gain*, which takes into account the fact that the measurements are not perfect. The gain at time 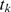 is given by:
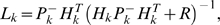
(7)where the matrix 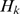 is the Jacobian of 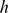 evaluated at the previous a posteriori state estimate. Given that, the current a posteriori state and error covariance estimates, denoted 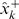 and 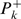 respectively, are formed using the following equations:
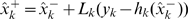
(8a)

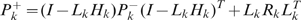
(8b)


We refer to [29] for a rigorous derivation of the equations we presented so far.

We remark that the algorithm we just introduced, as well as the ones employed in other works [25]–[27], provides *unconstrained* estimates. In some cases it is necessary to take into account equality or inequality constraints that prevent 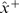 from assuming certain values. This can be important for the following reasons.

1. To incorporate into the estimation process prior knowledge that might be available on some of the quantities in the model.
2. To keep the estimates biologically meaningful. Depending on how the model is formulated, certain quantities may be sign-definite. In many cases, for example, both the states and the parameters must be positive.
3. To ensure that the evaluation of the functions 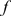 and 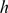 and of their Jacobians 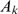 and 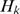 are well-posed. The algorithm will not work if at any given step 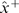 lays outside the domain of definition of 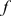 and 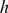 or of their partial derivatives.

To cope with these issues, we apply the constrained estimation technique developed in [30],[31]. This is derived using the fact that the estimate 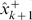 is the value that maximizes the conditional probability of 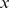 given the measurements 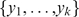 up to time 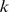. Furthermore, 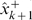 and 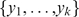 are jointly Gaussian, which means that 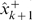 is conditionally Gaussian given 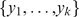. Finally, if 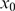, 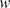 and 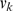 are jointly Gaussian, then 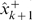 is the conditional mean of 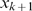 given the measurements 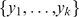. These three properties, which are derived in [32], imply that the conditional probability of 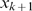 given 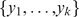 can be written as:
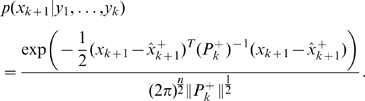
Now, suppose we have a set of linear constraints of the form 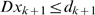, where 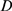 is a constant matrix of suitable dimensions. If 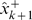 does not satisfy the constraints, we need to replace it with a constrained estimate 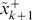. This can be obtained by maximizing 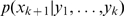 subject to the constraints, or equivalently, by maximizing its natural logarithm. Therefore, the problem we need to solve can be cast as:
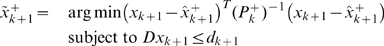
(9)


Since 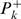 is a covariance matrix, and it is therefore strictly positive definite, this is a *strictly convex* quadratic programming problem that can be easily solved using standard algorithms, such as reflective Newton methods [33] and active set methods [34].

### Constrained HEKF algorithm summary

1. Set the initial conditions according to (4) and (5).
2. Compute the Jacobians of 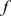 and 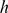 around the previous a posteriori state estimate.
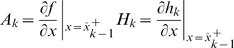
(10)
3. Advance to the next time step using (6).
4. Compute the gain using (7)
5. Incorporate the current measurement using (8).
6. Check if the estimate satisfies the constraints. If not, replace it with the solution of (9).
7. Repeat steps 2–6 for all the time instants 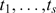.

### An a posteriori identifiability test

While for linear models the Kalman filter has nice convergence properties, in the case of the *extended* Kalman filter for nonlinear systems no such properties have been proven yet. As it is well-known in the literature [29], sometimes the filter may diverge, or may give biased estimates. While the first situation is easily detected in any implementation, the second one is dangerous, because the algorithm appears to run normally but produces severely wrong results. It is therefore extremely important to have a test that allows us to assess the reliability of the estimates.

The test we present here is based on a simple estimation of the variance of a random variable. Consider again a continuous-time process which is measured at discrete time instants. Assuming we are able to measure 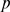 different quantities, we can rewrite our model expanding the 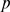 components of the output:
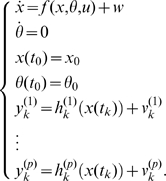
(11)As in the previous Section, we assume that 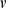 is a Gaussian random variable with zero mean and diagonal covariance matrix 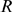. This means that 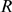 is a 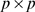 matrix, whose diagonal entries 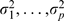 are the variances of each component of 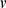. What (11) says is that each output is a sampled version of the corresponding function of the state, with an additive noise superimposed to it.

Now, suppose that by running the HEKF we find an estimate 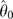 of 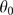. Let 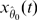 be the solution of (11) corresponding to 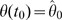. If we accept 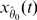 as a good approximation of the real solution 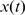, then we can write estimates of each component of the noise as:
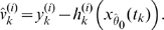
(12)This equation, for 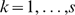, gives 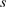 samples of 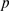 Gaussian random variables with zero mean. The main idea behind the test is that if 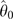 is close to 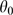, and consequently 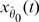 is close to 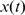, then the variance of 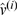 will be close to the variance of 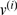.

Let 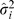 be the variance of 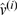. We can use the samples (12) to build a *point estimate* of 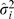 in the following way:
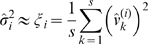
(13)The random variable 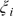 has a probability density function equal to the 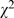 distribution with 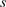 degrees of freedom [35].

Using this fact, we can form *interval estimates* of 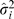 corresponding to different confidence coefficients 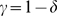. The confidence coefficient is a probability, so it takes values between 0 and 1. Common values for 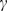 include 0.9, 0.95 and 0.997. Denote by 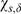 the 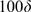-th percentile of the 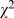 distribution with 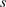 degrees of freedom. Then, 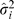 is in the interval
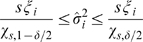
(14)with a probability of 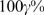.

It is then clear that if the real variance 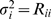 of 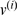 does not lie in the interval indicated by (14), it is extremely unlikely that the measurements 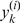 were generated by the set of parameters 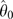, given the fact that the noise 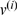 has a variance of 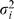. Therefore, we can reject the estimate 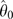 as wrong with a confidence of 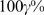.

We remark that this test can be also used independently of the HEKF to validate/invalidate the estimates computed by any other parameter estimation method.

### Estimate refinement

Although the HEKF can be applied to fairly large extended systems, when the parameter space is very large (and the extended system is therefore not observable) a single run of the filter will generally yield estimates that do not satisfy the 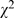 identifiability test described in the previous Section. Also, the estimates will be characterized by large uncertainties, as one can see by inspecting the entries of the 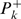 matrices. In this situation, the solution to the parameter estimation problem is not unique, therefore there will be infinite sets of parameters that are all equally likely to be correct. The best that one can do in this case is to find one or more values of 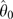 such that the corresponding solutions are consistent with the experimental observations in the sense of the 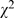 test.

In order to do that, we can make use of the probabilistic information we have about the measurement noise 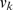. In particular, we know that 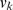 is a Gaussian random variable with zero mean and covariance 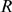. As we saw in the previous Section, given a certain estimated parameter set 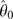, we can construct 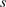 samples of an estimate 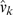 of 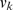 through (12). It makes sense, then, to ask for which values of 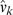 the mean and variance of 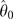 will be close to zero and 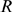 respectively. In other words, one can *minimize* the expected value 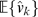 and the difference between 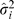 and 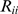 by solving the following problem:
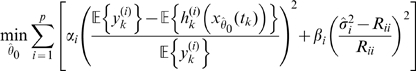
(15)


The weights 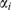 and 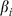 can be chosen by the user to attribute different relative importances to the mean matching and to the variance matching parts of the cost function. The most appropriate choice can be different for different problems. Note the scaling that is introduced in the function, which ensures that all the measurements are equally weighted in the minimization process, regardless of their size. This problem will not have any special properties in general, so it can be solved with any general purpose minimization algorithm. The Broyden-Fletcher-Goldfarb-Shanno (BFGS) algorithm [36] has proven to be a good practical choice.

We argue that this *moment matching* optimization is a better alternative than directly fitting the data points, as it guarantees that the result of the optimization process will be a statistically valid parameter set in the sense of the 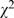 test (see the examples in the Results section).

**Figure 1:**
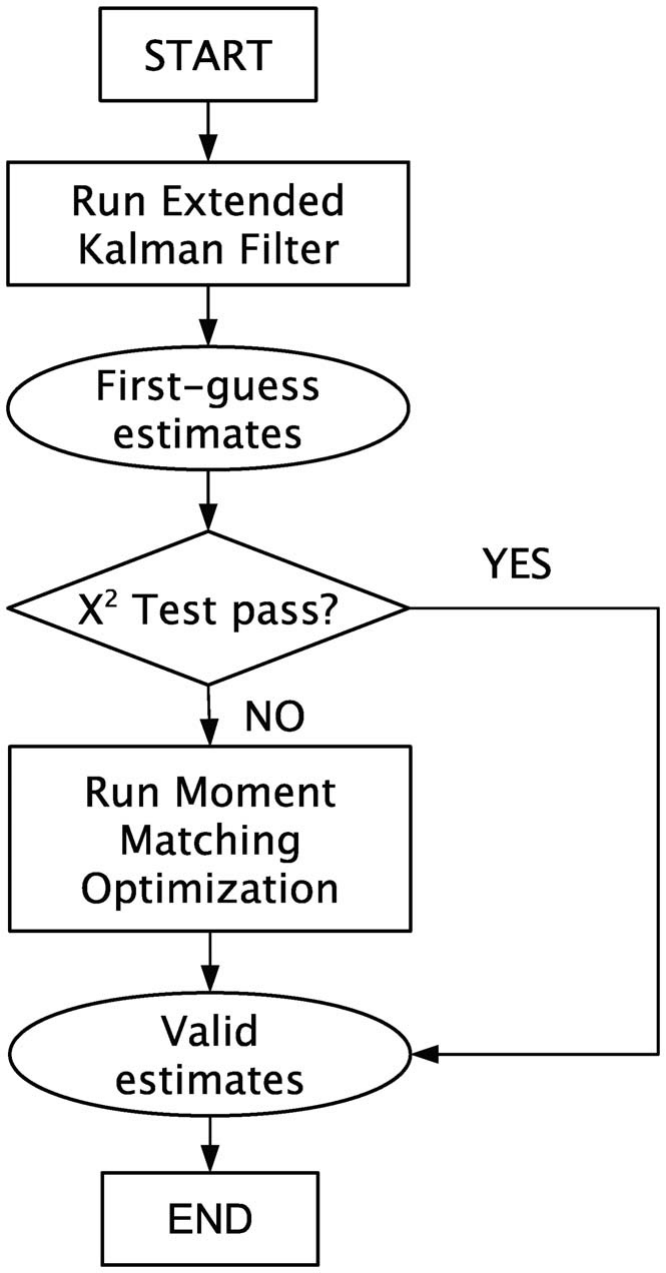
Flowchart of the proposed method. The algorithm is a three-stage process, which involves Kalman filtering, a statistical accuracy test and an optimization problem.

To summarize, the proposed algorithm is a three-stage process. In the first stage, we run the constrained HEKF algorithm on the model to get a first estimate of the unknown parameters. In the second stage we study a posteriori the identifiabilty problem, by running the 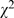 test. If the test is passed, the HEKF was able to recover the unique solution to the problem and the first estimate can be considered valid. If not, most likely no unique solution exists, and the first estimate needs to be refined by running the third stage, i.e. the moment matching optimization. The whole procedure is visualized in the flowchart of Figure 1.

### Model selection

One of the most interesting features of approaching the parameter estimation problem using state extension is that it allows for a *simultaneous estimation* of both the state and the parameters of the process under investigation. Therefore, the Kalman filter, together with the 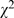 variance test we described, can also be used to address the problem of *model selection*.

Frequently, the structure of biochemical pathways is not completely known. One has an idea of the genes and proteins that play a role in a certain process, but the exact interconnections among such components are not fully elucidated. It may not be clear, for example, whether a certain gene is regulated using a positive feedback loop or a negative feedback loop, or if a certain reaction takes place with or without intermediate steps. In these scenarios, it is possible to write down different models corresponding to the different hypotheses and then use the Kalman filter to assess which one is the most likely to have generated the measurements that are observed in the experiments.

In order to simplify the presentation, suppose we have two different models of the form (3) for the same process. We can write them as:
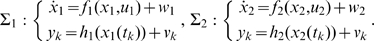
(16)The two models differ in everything except the measured data points 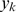 and the statistics of the noise 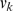 that is superimposed to them.

Running the HEKF for these models will give estimates of their states, which we will denote 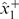 and 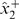. In analogy to what we did for the 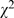 test, we can plug the estimates into 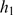 and 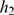 respectively. This will give two different estimates of the measurement noise 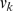:
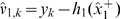
(17a)

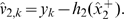
(17b)We can now form point estimates and interval estimates of the variance of each component of 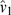 and 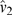 using (13) and (14) respectively. Again, the main idea behind this test is that the estimated variances that are closer to the real variances of the measurement noise 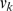 must come from the model that is more likely to have generated the measurements observed in the experiments. Moreover, if the real variances of 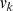 do not lie in the interval estimates computed for a certain model, we can reject that model as wrong with a probability of 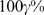, where 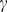 is the confidence coefficient that was used for the test.

Note that the two estimates of the measurement noise (17) can also be formed by using the model solution. However, using the Kalman filter estimates of the states allows the procedure to be carried out even if the initial conditions are unknown.

#### Model selection algorithm summary

1. Run the constrained HEKF on the models 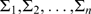 to get their state estimates 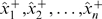.
2. Compute the estimates of the measurement noise 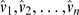 using (17).
3. Form point and interval estimates of the variance of each component of 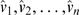 using (13) and (14).
4. Discard the models for which the interval estimates do not contain the real variances of 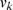.
5. Select the model whose variances match the best with the real variances of 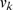.

## Results

### A model of the heat shock response in *E. coli*

#### The model

Exposure to high temperatures cause proteins to unfold from their functional three-dimensional structure. Misfolding can eventually result in the death of the cell. To mitigate the deleterious effects of heat, cells express heat-shock proteins whose role is to refold unfolded or misfolded proteins.

In *E. coli*, the heat shock response is implemented through an intricate architecture of feedback loops centered on the sigma-factor that regulates the transcription of the heat shock proteins under normal and stress conditions. The enzyme RNA polymerase (RNAP) bound to this regulatory sigma-factor, 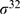, recognizes the heat shock gene promoters and transcribes specific heat shock genes. The heat shock genes encode predominantly molecular chaperones (i.e. enzymes that are involved in refolding denatured proteins), and proteases that degrade unfolded proteins. At physiological temperatures (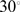
C to 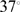
C), there is very little 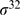 present and, hence, the levels of the heat shock genes are very low. When bacteria are exposed to high temperatures, 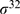 first rapidly accumulates, allowing increased transcription of the heat shock genes and then declines to a new steady state level, characteristic of the new temperature. The accumulation of high levels of heat shock proteins leads to the efficient refolding of the denatured proteins, thereby decreasing the pool of unfolded protein.

The following reduced order model of this process has been developed by El-Samad et al. [37].
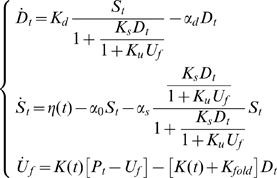
(18)In this model 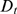 represents the number of molecules of chaperones, 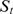 the number of molecules of the factor 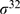, and 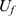 the total number of unfolded proteins. For further details on the model and the value of the parameters, see [37] and the references therein.

#### Small parameter space case

To demonstrate the use of the ideas we described in the Methods Section, suppose we want to estimate the parameters 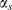 and 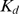 in (18). We assume that measurements of the variables 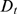 and 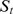 are available. The measurements are assumed to be very noisy and sparse.

As soon as the temperature is increased, we observe a rapid accumulation of the chaperones and of the 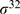 factor. After approximately 50 minutes, the system reaches a new steady state, characterized by elevated levels of these proteins. Given this kind of behavior, it makes sense to take measurements very frequently soon after the heat shock is applied. The sampling interval can then be increased, since the system doesn't evolve as quickly any more. We choose to sample at 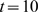, 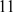, 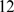, 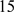, 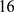, 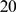 and 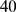 minutes. From 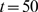 to 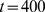 we choose a constant sampling period of 25 minutes. This choice requires the collection of 22 total data points.

**Figure 2:**
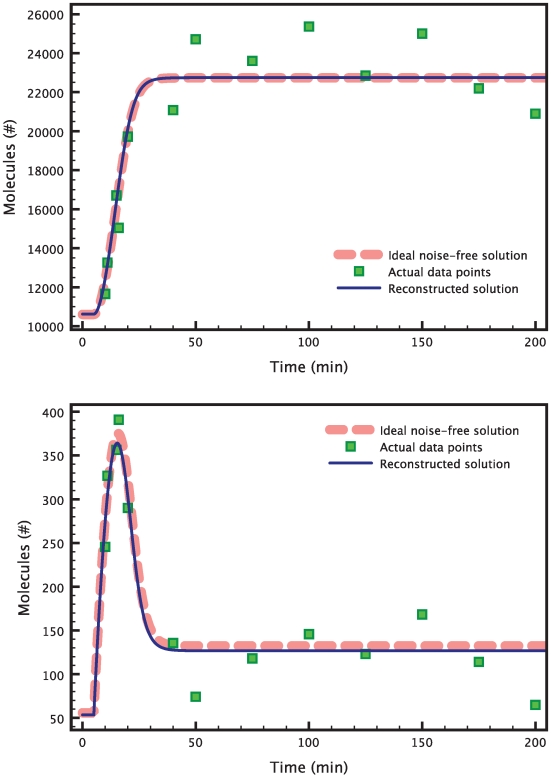
Estimation of 2 parameters in the heat shock model. The data points (green squares) are obtained by evaluating the true model solution (red dashed curve) at the chosen time points, and then adding white Gaussian noise. The blue solid line shows the reconstructed solution corresponding to the HEKF estimates for the parameters 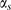 and 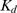. Both the reconstructed measurement signal for 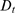 (top) and the one for 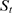 (bottom) are very close to the respective true solutions. The graphs are zoomed to highlight the transient response of the heat shock system after a temperature increase.

Once the time vector has been determined, we can run the experiments and collect our measurements. In this example, the data for the measurements are generated *in silico*. First we simulate the model and evaluate the solution at the given time instants, and then we add white Gaussian noise to it to simulate measurement noise. Typical measurement signals are shown in Figure 2. The components of the noise have variances equal to 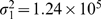 and 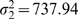. The red dotted lines represent the noise-free solutions obtained from the run of the model. The green squares represent the actual information known to the filter. The data points are collected at the sampling instants described above.

**Figure 3:**
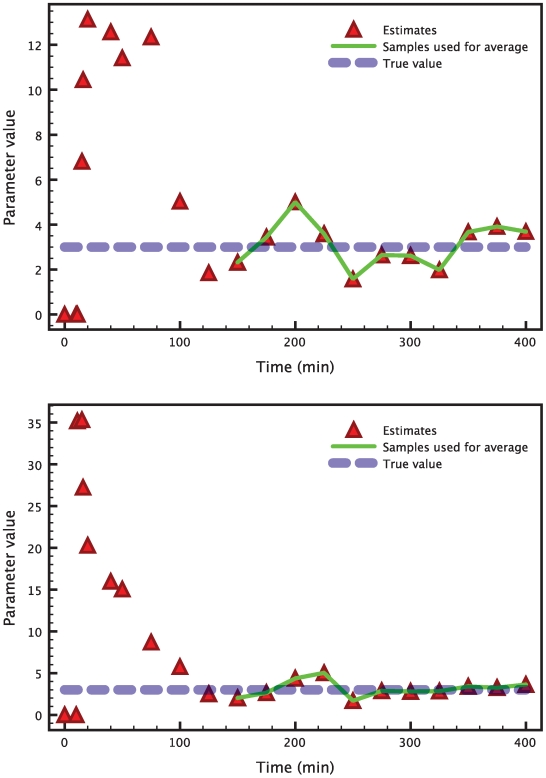
Time evolution of the Kalman filter parameter estimates in the heat shock model. After an initial transient, the estimates of the two parameters 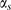 (top) and 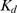 (bottom), represented by the triangles, keep oscillating around the respective true values (blue dashed line). The last 10 samples (connected by the green line) are averaged to extract a single number from this time-varying signal.

Figure 3 shows the results of a typical run of the hybrid extended Kalman filter applied to this problem. The filter is started from initial conditions equal to 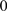. The dotted line represents the true value of the parameter. The red triangles show how the filter updates the estimate based on the information that comes from the measurements. After a transient, the estimates keep oscillating around the true values of the parameters. From this time-varying signal, a single number is extracted by averaging over the last ten samples (marked by the green line), when the filter has converged to a steady state. The final estimates for this simulation are
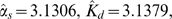
while the true value is 3 for both parameters. Even in presence of such high levels of noise, the estimation is very accurate, with less than 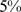 error. The ideal and reconstructed solutions are almost indistinguishable.

#### A posteriori identifiability test

To check the estimation results we just obtained, we compute the point and interval estimates of the variances of the two components of the measurement noise according to (13) and (15) respectively. We fix a confidence coefficient of 0.95.

For the first component of the noise we get
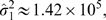
and
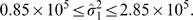
The error between the real variance 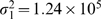 and the point estimate is only 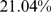. Moreover, 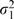 lies in the interval estimate.

For the second component of the noise we get
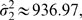
and
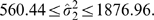
The error between the real variance 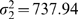 and the point estimate is only 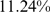. Moreover, 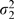 lies in the interval estimate. These results confirm that the estimates we obtained using the hybrid extended Kalman filter can be considered valid.

#### Model selection

**Figure 4:**
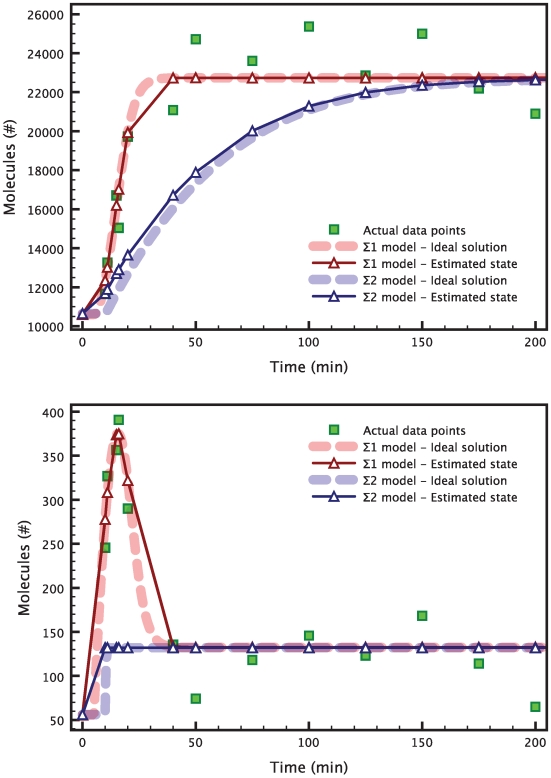
Discrimination between competing heat shock models. The models (18) (blue) and (19) (red) are compared in terms of their 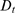 (top) and 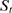 (bottom) outputs. Both signals evolve to the same steady state, but with different transient behavior. The dashed lines represent the ideal model solutions, the triangles are the corresponding Kalman filter estimates.

To illustrate the use of the Kalman filter for model selection, consider again the measurements signals shown in Figure 2. In this case, the problem is not the estimation of the parameters (which are assumed to be known), but the comparison of two different models for the process. Following the notation introduced earlier, let 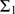 be (18) and 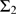 be the model:
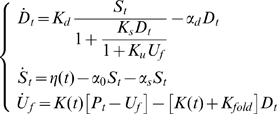
(19)The key difference between 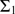 and 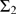 is the presence or absence of the spike in the 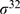 factor following the heat shock. This corresponds to turning off one of the feedback loops in the heat shock response system. The two solutions are compared in Figure 4. The thick dotted lines in the plots represent the ideal time evolutions of chaperones and 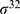 factor simulated using 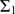 (red) and 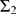 (blue). The triangles represent the relative estimated temporal evolutions using the HEKF.

We now obtain the estimates of the measurement noise using (17) and compute the point and interval estimates of the variances of their components using (13) and (14). The results are summarized in Table 1. It is clear that only 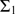 produces results that are compatible with the measurements. Therefore, we can reject 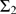 as an inaccurate model with a probability of 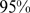.

**Table 1:** Discrimination of the heat shock models.

Model	Component 1		Component 2	
	Point	Interval	Point	Interval
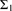	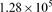	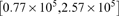	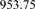	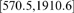
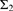	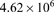	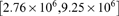	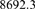	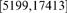
Real variances	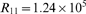		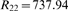	

The table shows the point estimates (13) and interval estimates (14) of the measurement noise variances corresponding to the models 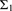 and 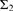. We note that the real variances encoded in the matrix 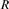 lie inside the interval estimates for 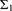, but not inside the ones for 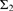. The 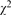 test indicates that only 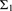 is consistent with the data.

#### Large parameter space case

Suppose now we seek to estimate 6 of the parameters in (18), namely 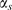, 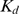, 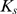, 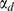, 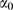 and 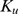. We are going to use the same type of measurements as in the previous case, with as many data points and as much noise.

**Figure 5:**
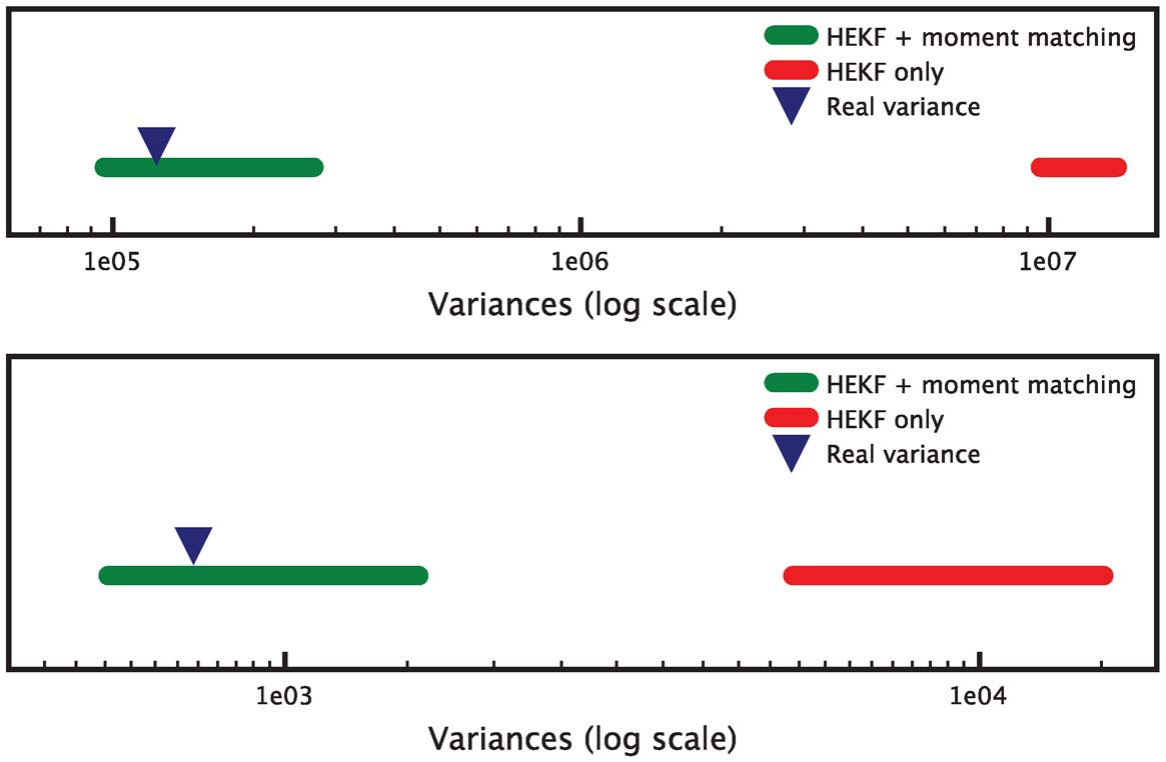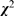 interval estimates in the case of valid and invalid parameter sets. The red set of interval estimates corresponds to a parameter set computed with the HEKF only (invalid). The green set corresponds to a parameter set that was obtained with the combination of HEKF and moment matching optimization (valid). The real variances (blue triangles) only lie inside the intervals corresponding to a valid estimation. The top panel is relative to the 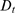 measurement signal, the bottom panel to the 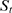 measurement signal.

In this new example, a single run of the HEKF produces values that do not satisfy the 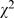 identifiability test. The interval estimates generated by the test do not contain the real variances that were used to generate the measurements, thereby indicating that the parameter values inferred by the HEKF can not be considered valid (Figure 5). Therefore, we apply the estimate refinement technique introduced in the Methods Section. We minimize (15) with 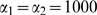 and 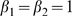. For the minimization we use the Broyden-Fletcher-Goldfarb-Shanno (BFGS) algorithm, as described in [36] and as implemented in the GNU Scientific Library [38].

**Figure 6:**
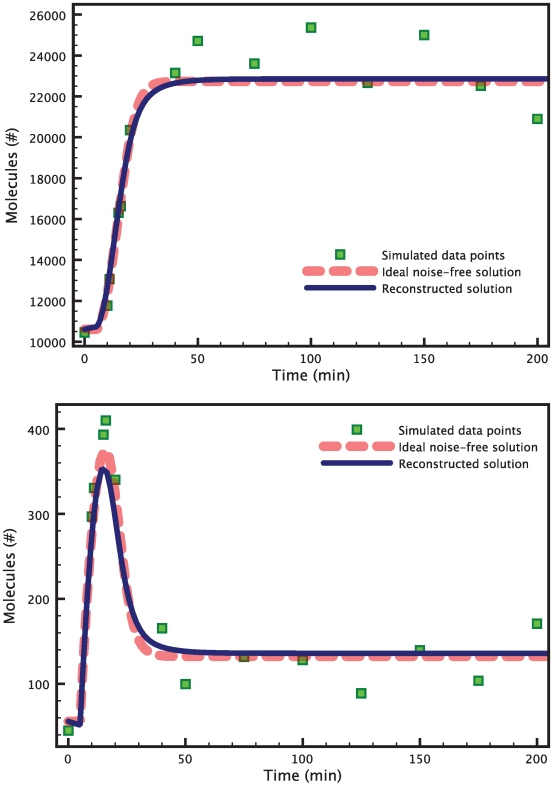
Estimation of 6 parameters in the heat shock model. The data points (green squares) are obtained by evaluating the true model solution (red dashed curve) at the chosen time points, and then adding white Gaussian noise. The blue solid line shows the reconstructed solution corresponding to the parameters estimates. Both the reconstructed measurement signal for 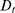 (top) and the one for 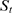 (bottom) are very close to the respective true solutions. The graphs are zoomed to highlight the transient response of the heat shock system after a temperature increase.

The results are presented in Figure 6. A minimum was found after 1560 iterations of the BFGS algorithm, with a cost value of 0.14 (note that the optimal value of the cost is zero). The minimization took about 3 hours to run on a MacBook Pro with a single 2.6 GHz processor. Figure 5 shows how the interval estimates of the variances for the refined estimates now contain the real variances.

We also compared the results of our method with a nonlinear Levenberg-Marquardt least-squares fitting and a genetic algorithm fitting directly on the data points. The results are summarized in Tables 2, 3 and 4. If we compare the three tables, it is clear that only our method was capable of estimating a parameter set that was consistent with the simulated data in the sense of the 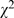 test.

**Table 2:** 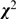
 test results for the estimation of 6 parameters in the heat shock model (moment matching).

Method	BFGS moments	
	Component 1	Component 2
Mean error	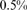	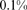
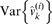 point	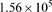	890.6
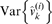 interval	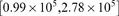	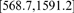
Real variances	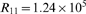	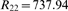
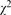 test result	pass	

The table shows the point estimates (13) and interval estimates (14) of the measurement noise variances corresponding to the parameter set computed using the BFGS moment matching optimization described in the Methods Section. The optimization took 1560 iterations (about 3 hours running time). All the interval estimates contain the corresponding real variances, indicating that the parameter set can be considered valid in the sense of the 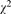 test.

**Table 3:** 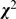
 test results for the estimation of 6 parameters in the heat shock model (nonlinear least-squares).

Method	LM data	
	Component 1	Component 2
Mean error	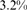	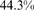
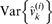 point	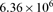	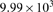
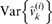 interval	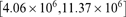	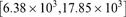
Real variances	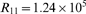	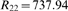
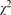 test result	fail	

The table shows the point estimates (13) and interval estimates (14) of the measurement noise variances corresponding to the parameter set computed using a nonlinear least-squares fitting directly on the data points. The fitting was carried out with the Levenberg-Marquardt algorithm (LM). The optimization took 115 iterations (about 21 minutes running time). The interval estimates do not contain the corresponding real variances, indicating that the parameter set is invalidated by the 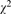 test.

**Table 4:** 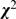
 test results for the estimation of 6 parameters in the heat shock model (genetic algorithm).

Method	GA data	
	Component 1	Component 2
Mean error	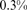	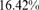
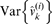 point	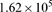	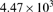
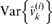 interval	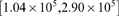	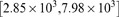
Real variances	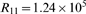	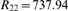
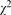 test result	fail	

The table shows the point estimates (13) and interval estimates (14) of the measurement noise variances corresponding to the parameter set computed using a genetic algorithm (GA) fitting directly on the data points. The optimization took 106 iterations (about 5 minutes running time). The interval estimate for component 2 does not contain the corresponding real variance, indicating that the parameter set is invalidated by the 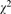 test.

### The repressilator

The repressilator is a synthetic gene regulatory network, whose model is frequently used as an example for numerical algorithms [10],[27]. It consists of three genes connected in a feedback loop, where each gene transcribes the repressor protein for the next gene in the loop. The original model of Elowitz and Leibler [39] consists of six equations with four parameters, where all the three genes have the same production and degradation rates, and are affected in the same way by the corresponding repressor. Likewise, the three proteins have the same production and degradation rates.

In this example we consider a more general version of the repressilator, where each component is allowed to have different parameters. The model equations are as follows
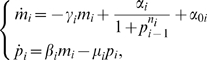
(20)for 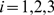, with the convention that 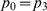. The interactions of each gene/protein pair are characterized by 6 rates, therefore the total number of parameters to be estimated is 18.

We are assuming that we are able to measure the mRNA concentrations (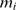), but not the protein concentrations (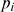). We collect 30 equally spaced data points for each mRNA species. The noise in the measurements is assumed to have a power (i.e. variance) of 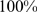 of the mean of the signal. The parameters and the initial conditions to generate the simulated data are chosen so that the system displays a limit cycle behavior.

**Figure 7:**
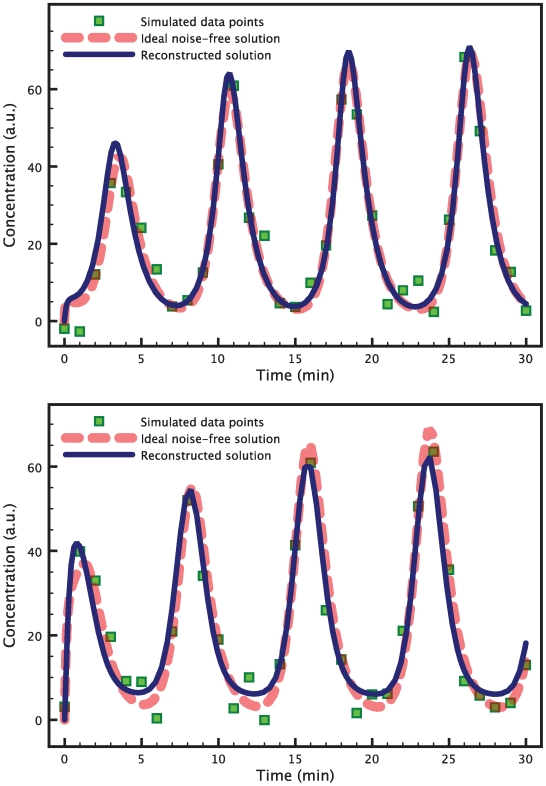
Estimation of 18 parameters in the repressilator model. The data points (green squares) are obtained by evaluating the true model solution (red dashed curve) at the chosen time points, and then adding white Gaussian noise. The blue solid line shows the reconstructed solution corresponding to the estimated parameters. Both the reconstructed measurement signal for 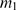 (top) and the one for 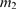 (bottom) are very close to the respective true solutions. The graph for the measurement 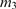 is presented in the supporting Figure S1.

As in the large parameter space case for the heat shock model, a single run of the HEKF produces estimates that do not satisfy the 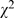 identifiability test. Therefore, we apply the estimate refinement technique by minimizing (15) with 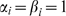. The results are presented in Figure 7. For the sake of brevity, we only show the 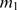 and 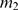 measurements. The 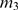 measurement is presented in the supporting Figure S1.

We also compared the results of our method with a nonlinear Levenberg-Marquardt least-squares fitting and a genetic algorithm fitting directly on the data points. The results are summarized in Tables 5, 6 and 7. Only our method was capable of estimating a parameter set that was consistent with the simulated data in the sense of the 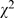 test.

**Table 5:** 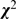
 test results for the estimation of 18 parameters in the repressilator model (moment matching).

Method	BFGS moments		
	Component 1	Component 2	Component 3
Mean error	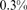	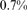	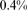
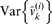 point	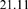	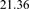	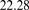
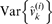 interval	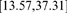	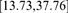	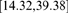
Real variances	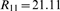	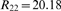	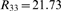
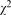 test result	pass		

The table shows the point estimates (13) and interval estimates (14) of the measurement noise variances corresponding to the parameter set computed using the BFGS moment matching optimization described in the Methods Section. The optimization took 720 iterations (about 26 minutes running time). All the interval estimates contain the corresponding real variances, indicating that the parameter set can be considered valid in the sense of the 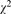 test.

**Table 6:** 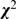
 test results for the estimation of 18 parameters in the repressilator model (least-squares).

Method	LM data		
	Component 1	Component 2	Component 3
Mean error	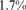	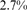	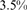
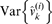 point	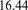	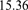	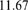
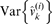 interval	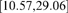	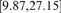	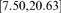
Real variances	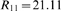	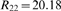	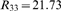
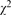 test result	fail		

The table shows the point estimates (13) and interval estimates (14) of the measurement noise variances corresponding to the parameter set computed using a nonlinear least-squares fitting directly on the data points. The fitting was carried out with the Levenberg-Marquardt algorithm (LM). The optimization took 129 iterations (about 3 minutes running time). The interval estimate for component 3 does not contain the corresponding real variance, indicating that the parameter set is invalidated by the 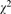 test.

**Table 7:** 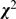
 test results for the estimation of 18 parameters in the repressilator model (genetic algorithm).

Method	GA data		
	Component 1	Component 2	Component 3
Mean error	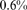	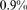	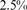
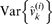 point	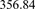	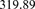	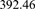
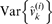 interval	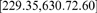	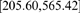	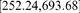
Real variances	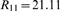	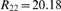	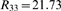
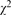 test result	fail		

The table shows the point estimates (13) and interval estimates (14) of the measurement noise variances corresponding to the parameter set computed using a genetic algorithm (GA) fitting directly on the data points. The optimization took 101 iterations (about 4 minutes running time). The interval estimates do not contain the real variances, indicating that the parameter set is invalidated by the 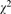 test.

## Discussion

We have presented a novel approach for parameter estimation and model selection in computational biology. We have used this approach as a basis for a new algorithm for estimating parameters in models of biological systems from noisy and sparse experimental measurements. The approach is based on the combination of an extended Kalman filter algorithm, a statistical accuracy test, and a moment matching procedure. Furthermore, we have showed how the same tools can be used to discriminate among different candidate models of the same biological process. We have demonstrated the application of these ideas through two examples, a reduced order model of the heat shock response in *E. coli* and a generalized model of the repressilator (an additional example is available in the supporting file Text S1).

Parameter estimation using state observers in general, and the Kalman filter in particular, confers the significant advantage of fully exploiting the prior knowledge on the process that is encoded into the model. Observers are designed using the system's equations themselves, thus taking into account the system's dynamics. The Kalman filter has nice properties that are guaranteed to hold when the underlying dynamical system is linear and the noise statistics are Gaussian. In this case, the Kalman filter is the *optimal* state estimator, meaning that it produces the estimates with the smallest standard deviation of the estimation error. Even if the noise is not Gaussian, the Kalman filter is the optimal linear estimator. When the filter is extended for use with nonlinear dynamical systems through the time-varying linearization (10), such properties only hold in an approximate way, and one loses many of the theoretical guarantees that apply when the model is linear. However, in practice the extended Kalman filter has proven to be a successful choice in a wide range of applications, becoming the de-facto standard in nonlinear state estimation [29].

The Kalman filter approach to parameter estimation displays some features that make it particularly well suited to biological applications. For example, the *hybrid* Extended Kalman Filter (HEKF) is capable of estimating the parameters of continuous-time models with discrete-time measurements. This is important because most deterministic models of biological systems are continuous-time. However, most experimental techniques produce discrete-time data, often with large and non-uniform sampling intervals. The presented algorithm accommodates such situations without introducing any additional error due to a discretization of the system equations.

In spite of the above advantages, several challenges arise when using the Kalman filter for parameter estimation in a general nonlinear model. First, in the nonlinear setting, the Kalman Filter is not in general the optimal estimator. Moreover, if the initial estimates are too far off the filter may diverge, or converge to an estimate whose mean is different from the true mean. Additional factors can also be a source of error. State observers, as the name implies, were originally developed to estimate the *state* of a system – not its parameters. The state extension that becomes necessary to include the parameters into the estimation variables can introduce non-uniqueness of the solution (loss of observability), which can be problematic for the algorithms [29]. Furthermore, the covariance propagation equation in (6) is subject to numerical ill-conditioning, which can make the estimated error covariance matrices unreliable. These are some of the key reasons why the extended Kalman filter can produce unreliable estimates, and consequently, why a refined algorithm is needed for parameter estimation.

To alleviate some of the shortcomings of the HEKF in parameter estimation, we have proposed to augment the HEKF with an a posteriori 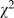 statistical test and a subsequent optimization stage, both of which explicitly incorporate the information about measurement noise statistics into the estimation process. The test serves as a tool for the statistical reliability assessment of computed estimates, which validates the consistency of these estimates with respect to noise statistics. It also inspires a new technique for the discrimination between different candidate models for the same process. When the 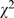 test shows that filter parameter estimates are inconsistent with the noise model, which can happen for any of the reasons mentioned in the previous paragraph, an estimate refinement step can become necessary. This takes the form of an optimization stage that begins where the HEKF left off. This proceeds until an estimate that satisfies the 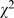 test is reached.

If the 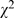 test for parameter estimates is the sole measure for accepting or rejecting a parameter estimate, then why not use it solely for parameter estimation by optimizing that measure directly, bypassing the Kalman Filter altogether? In the small parameter space case, numerical evidence suggests that if a unique solution to the parameter estimation problem exists, the HEKF is able to infer it with great speed and accuracy. This was seen in both the heat shock model and in the gene expression model, available in the supporting file Text S1. If the number of parameters is large and a good initial guess is not available, the HEKF is still able to run and provide a suitable initial guess for the subsequent refinement step, which can be expected to significantly reduce the running time of the moment matching optimization. Furthermore, the HEKF provides a computationally cheap algorithm, which scales much better than e.g. Bayesian methods and the particle filter. For these reasons, we believe that the HEKF represents a good choice as a first stage followed by moment matching optimization.

Coupling the Kalman filter with the statistical moment matching minimization presents a new way of thinking about optimization in parameter estimation. Classically, optimization is performed by trying to fit the model solution with the experimental data. While this is successful in some cases, it gives no guarantee that the parameters will produce a solution that is statistically consistent with the data. In the repressilator example, for instance, the classical least squares fitting produces for the state 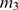 a variance that is too small compared to the one that was used to generated the simulated measurements (Table 6). In this situation, one runs into the issue of overfitting, in which the fitted model seems to replicate very well the behavior suggested by the data but fails to be robust to perturbations, so whenever it is used for further investigation, its behavior exhibits large inaccuracies. In contrast, the approach proposed here aims to match the mean and the variance of the measurement noise instead of the data points themselves, and is therefore able to “look beyond the noise” to recover the model parameters.

## Supporting Information

Text S1 Text supporting information file, with additional examples and discussion.
(0.29 MB PDF)

Figure S1 Estimation of 18 parameters in the repressilator model (measurement m_3_). The data points (green squares) are obtained by evaluating the true model solution (red dashed curve) at the chosen time points, and then adding white Gaussian noise. The blue solid line shows the reconstructed solution corresponding to the estimated parameters.
(0.42 MB EPS)
