# Supplementary material for: Parameter Estimation and Model Selection in Computational Biology
Source: PLoS Comput Biol. 2010 Mar 5;6(3):e1000696. doi: 10.1371/journal.pcbi.1000696 (PMC2832681; doi:10.1371/journal.pcbi.1000696)
Supplement: Text S1 — Text supporting information file, with additional examples and discussion. (0.29 MB PDF) [file pcbi.1000696.s001.pdf]

Parameter estimation and model selection in computational biology

Gabriele Lillacci[1], Mustafa Khammash[1,*],
1 Center for Control, Dynamical Systems and Computation, University of California at Santa Barbara, Santa Barbara, CA, USA
∗ E-mail: khammash@engr.ucsb.edu

Supplementary material

A simple model of gene expression

The model

The life of every organism is ultimately controlled by gene expression, the process by which the genetic information stored in its DNA is used to synthesize functional products such as RNA and proteins. This process involves two main steps, transcription and translation. In many cases a protein does not interact with other components of biochemical pathways unless it is somehow "activated" through a post-translational modification. A very common one is *phosphorylation*.

A simple dynamic model for transcription, translation and activation of protein through phosphorylation is given by the following set of ODEs:

$$\begin{cases} \dot{x}_1 = \lambda - \mu_1 x_1 \\ \dot{x}_2 = \alpha x_1 - \mu_2 x_2 + \nu x_3 - \kappa \dfrac{x_2}{1 + x_2} \\ \dot{x}_3 = -\nu x_3 + \kappa \dfrac{x_2}{1 + x_2}. \end{cases} \tag{1}$$

The state variables x_1, x_2 and x_3 represent concentrations of mRNA, protein and phosphorylated protein respectively. The model has six parameters with the following interpretation.

- λ is the transcription rate (μM min^{-1}).
- μ_1 is the spontaneous degradation rate of the mRNA (min^{-1}).
- α is the translation rate (min^{-1}).
- μ_2 is the spontaneous degradation rate of the inactive protein (min^{-1}).
- ν is the spontaneous dephosphorylation rate of the active protein (min^{-1}).
- κ is the speed of the enzymatic reaction leading to phosphorylation of the inactive protein (μM min^{-1}).

Note that in order to be biologically meaningful, all the state variables and the parameters have to be real and positive. This model has been introduced and studied in previous works as part of a deterministic modeling framework for biochemical networks [1, 2]. The parameters values we use for the simulations come from the model of the p53 network presented in [3].

Estimation of α and a posteriori identifiability test

In (1), the parameter α is of particular importance, because it gives a sense of how many copies of the protein are produced per single mRNA transcript. We will estimate this parameter using the HEKF. We suppose we are measuring protein concentration with an assay that is not able to distinguish among

different post-translational modification of the same protein. This is the case when, for example, one uses a non-specific antibody or a fluorescent marker. We choose a uniform sampling interval of 50 minutes and we collect 20 total data points from $t = 50$ to $t = 1000$. We assume that the measurement noise has a variance $\sigma^2 = 18.63$, which is equal to 100% of the mean of the measurement signal. The measurement signal is shown in Figure 1.

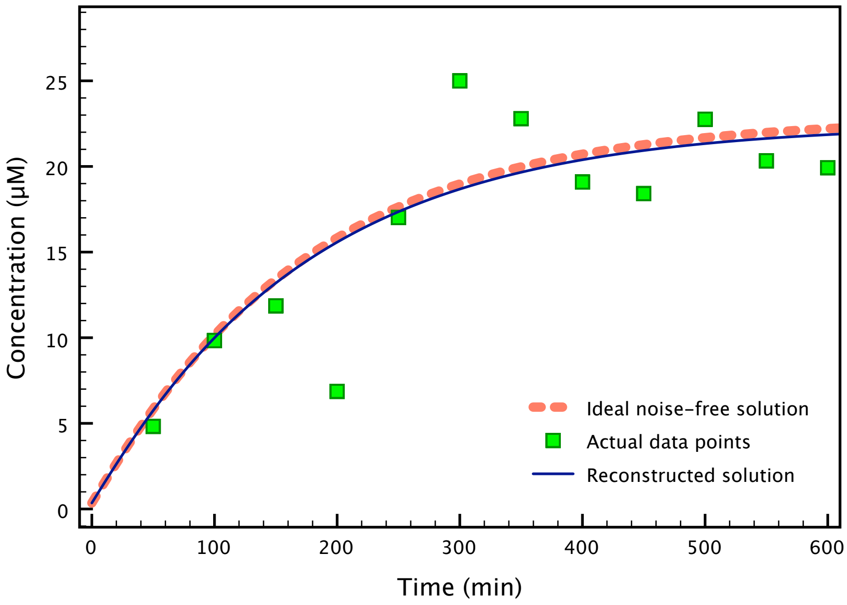

Figure 1. Estimation of α in the gene expression model. The data points (green squares) are obtained by evaluating the true model solution (red dashed curve) at the chosen time points, and then adding white Gaussian noise. The blue solid line shows the reconstructed solution corresponding to the HEKF estimates. The graphs are zoomed to highlight the transient behavior following the temperature increase.

Figure 2 shows the result of the estimation for a typical HEKF run. The filter is started from initial conditions equal to 0. The dotted line represents the true value of the parameter. The red triangles show how the filter updates the estimate based on the information that comes from the measurements. After a transient, the estimates keep oscillating around the true values of the parameters. From this time-varying signal, a single number is extracted by averaging over the last ten samples (marked by the green line), when the filter has converged to a steady state. The final estimated value for the simulation shown in the plot is

$$\hat{\alpha} = 0.1228,$$

while the true value of the parameter is 0.12. Also in this case, the estimation is very accurate, with less than 5% error.

Similar results hold for the estimation of the parameters λ, μ_1, μ_2. On the other hand, ν and κ cannot be estimated measuring $x_2 + x_3$. This can be seen with a simple observability analysis. In this case, we can say a priori that ν and κ are not identifiable.

To check the estimate we just obtained, we compute the point and interval estimates of the variances of the measurement noise as described in the Methods Section of the main paper. We fix a confidence coefficient of 0.95.

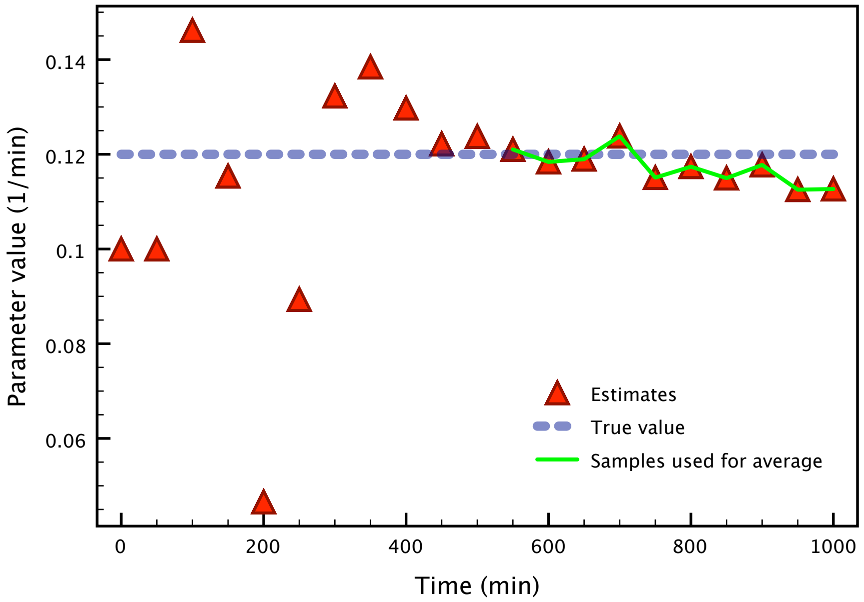

Figure 2. Time evolution of the α estimates in the gene expression model. After an initial transient, the estimates (triangles) keep oscillating around the true value (blue dashed line). The last 10 samples (connected by the green line) are averaged to extract a single number from this time-varying signal.

We get

$$\hat{\sigma}^2 \approx 23.55,$$

and

$$13.94 \leq \hat{\sigma}^2 \leq 48.01.$$

The error between the real variance $\sigma^2 = 18.63$ and the point estimate is only 26.40%. Moreover, σ_1^2 lies in the interval estimate. These results confirm that the estimates we obtained using the hybrid extended Kalman filter can be considered valid. The refinement step is not necessary in this example.

Suppose now the filter gave as results the values $\hat{\alpha} = 0.3$. The corresponding identifiability test gives

$$\hat{\sigma}^2 \approx 77.42,$$

and

$$45.83 \leq \hat{\sigma}^2 \leq 158.12.$$

We observe that the real variance is very far from its point estimate, and does not lie within its interval estimate. Therefore, we can reject the estimated parameter as incorrect with a probability of 95%.

Model selection

Many genes are regulated at the transcriptional level by the same protein they code for. This autoregulation motif is one of the most common observed in e.g. the genome of yeast, as reported in [4].

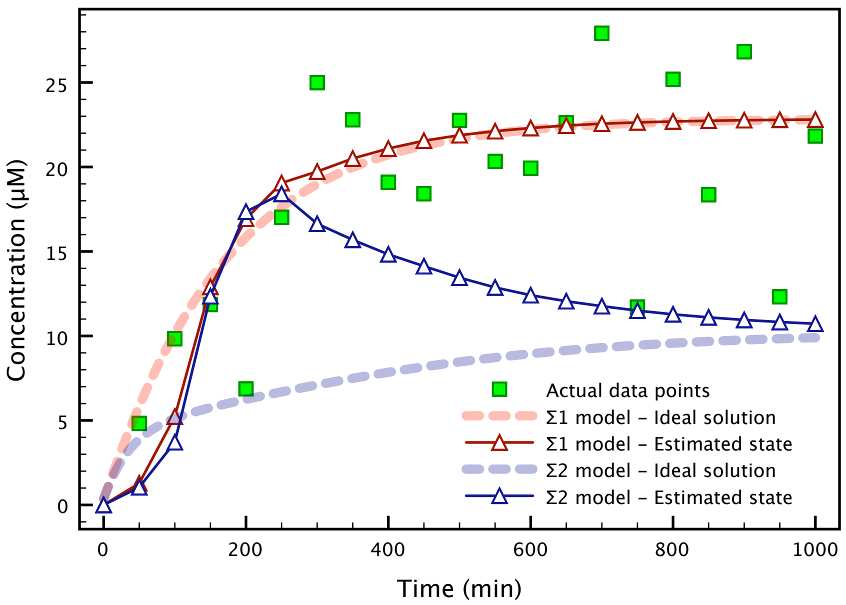

Figure 3. Discrimination between models (1) **(blue) and** (2) **(red).** The green squares represent the measurements. The dashed lines represent the ideal model solutions, the triangles are the corresponding Kalman filter estimates.

In this example, our goal is to find out whether the gene we are modeling displays autoregulation or not. Let Σ_1 be (1) and Σ_2 be the model:

$$\begin{cases} \dot{x}_1 = \lambda \dfrac{x_3^n}{K^n + x_3^n} - \mu_1 x_1 \\ \dot{x}_2 = \alpha x_1 - \mu_2 x_2 + \nu x_3 - \kappa \dfrac{x_2}{1+x_2} \\ \dot{x}_3 = -\nu x_3 + \kappa \dfrac{x_2}{1+x_2}. \end{cases} \tag{2}$$

The difference between the two models is that in Σ_2 the transcription rate λ is multiplied by a Hill-type function that represents the active protein feeding back into the mRNA and regulating its production. The two solutions are compared in Figure 3. The thick dotted lines in the plots represent the ideal time measurement signals with the two models. The triangles represent the reconstructed measurement signal using the HEKF.

We now obtain the estimates of the measurement noise and compute the point and interval estimates of its variance using the model selection algorithm described in the Methods Section of the main paper. The results are summarized in Table 1. It is clear that only Σ_1 produces results that are compatible with the measurements. Therefore, we can reject Σ_2 as an inaccurate model with a probability of 95%, and we can conclude that it is highly unlikely that the gene displays autoregulation.

Estimation performance and optimal sampling time of the HEFK algorithm

We next present a characterization of the estimation performance of the HEKF-based algorithm for the small parameter space case. Figure 4 shows performance statistics of the estimation of α_s and K_d in the heat shock model. These plots are formed by running a large number ($N = 10000$) of simulations

Table 1. Discrimination of the gene expression models.

Model	Variance estimates	
	Point	Interval
Σ_1	19.90	11.7813<br>40.65
Σ_2	41.69	24.68<br>85.14
Real variance	$R = 18.63$	

The table shows the point and interval estimates of the variances of the estimated measurement noise for the models Σ_1 and Σ_2. Comparing these quantities with the real variances encoded in the matrix R, it is clear that only Σ_1 is consistent with the data.

for different measurement signals and different values of the sampling time in the range from $t = 50$ to $t = 400$. From $t = 0$ to $t = 50$ the sampling instants are kept fixed to the values indicates earlier. The red squares show the mean over all the simulations of the estimates obtained in this way. The error bars represent an interval of ± 3 standard deviations around the mean (99.7% confidence interval).

The plots show the existence of an "optimal" sampling time that minimizes the standard deviation of the estimates. For the α_s, the optimal sampling time is 10 min, which corresponds to a standard deviation of 0.26. For the K_d, the optimal sampling time is 15 min, which corresponds to a standard deviation of 0.23. Therefore, when designing an experiment for this example, choosing one of these two values would be best. If this is not technically feasible, we can still use this kind of analysis to read the level of accuracy that we can expect using a given sampling time. For example, since we used 25 min, we can expect standard deviations is α_s and K_d of 0.28 and 0.24 respectively. Note that the optimal sampling time is not the smallest possible, as one would intuitively think. Rather, it represents a trade-off: if we use too few data points, the filter doesn't have enough information to produce accurate estimates, whereas if we use too many the accuracy degrades because we are introducing too much noise. Note also that using a sampling time greater than 40 min does not represent a good choice regardless of the standard deviation, because a significant bias is present in the estimates.

Figure 5 shows analogous performance statistics for the estimation of α in the gene expression model.

In this case, the optimal sampling time is 30 min, corresponding to a standard deviation of 0.009. Since we choose a sampling time of 50 min, we can expect a standard deviation of 0.01, which is still very close to the optimal value. Note how sampling times smaller than 15 min or greater than 90 min do not represent good choices, since they correspond to relatively high standard deviations or biased means.

We can get an even better quantitative idea of the performance of the algorithm by comparing the power (i.e. the variance) of the noise in the measurement signals to the variance of the estimates. This is easily done again using the plots in Figures 4 and 5. In the heat shock example, the variances of the components of the measurement noise are 1.24×10^5 and 737.94, which are respectively equal to 530% and 603% of the averages of the ideal noise-free solutions presented in Figure 2 of the main text. The variances of the estimates for α_s and K_d are 0.078 and 0.058 respectively, which are equal to only 2.6% and 1.9% of the true values of the parameters. In the gene expression example, the variance of the measurement noise is 18.63, which is equal to 160% of the average of the ideal noise-free measurement signal presented in Figure 1. The variance of the estimates for α is 0.0001, which is equal to 0.08% of the true value of the parameter.

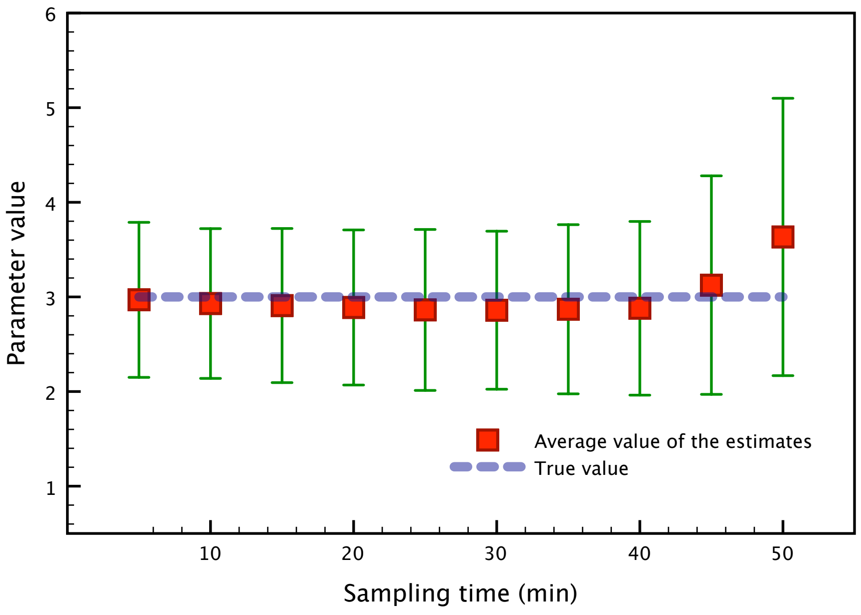

(a) α_s

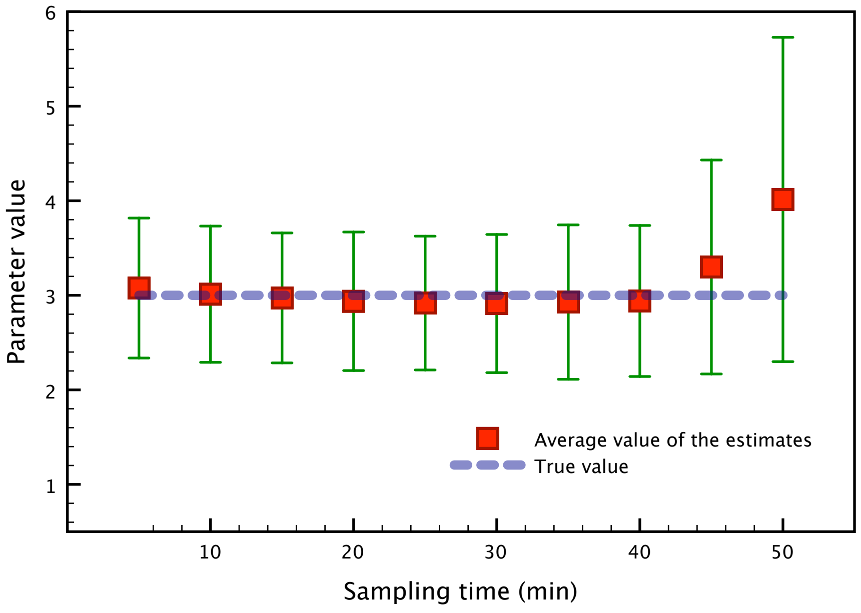

(b) K_d

Figure 4. Performance of the HEKF for the estimation of the α_s and K_d in the heat shock model. These plots are formed by running $N = 10000$ simulations for different measurement signals and different values of the sampling time. The red squares show the mean over all the simulations of the estimates obtained in this way. The error bars represent an interval of ± 3 standard deviations around the mean (99.7% confidence interval).

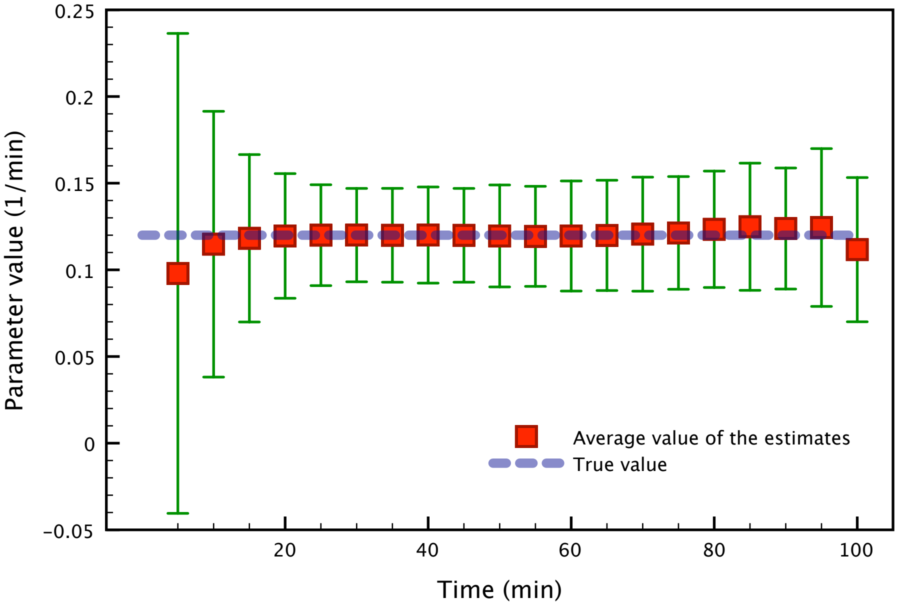

Figure 5. Performance of the HEKF for the estimation of the α in the gene expression model. These plots are formed by running $N = 10000$ simulations for different measurement signals and different values of the sampling time. The red squares show the mean over all the simulations of the estimates obtained in this way. The error bars represent an interval of ± 3 standard deviations around the mean (99.7% confidence interval).

References

1. Lillacci G, Valigi P (2008) State estimation for a model of gene expression. In: IEEE International Symposium on Circuits and Systems (ISCAS08). doi:10.1109/ISCAS.2008.4541850.

2. Lillacci G, Valigi P (2007) State observers for the estimation of mRNA and protein dynamics. In: 3rd IEEE-NIH Life Science Systems and Applications Workshop (LISSA07). pp. 108–111. doi:10.1109/LSSA.2007.4400896.

3. Lillacci G, Boccadoro M, Valigi P (2006) The p53 network and its control via MDM2 inhibitors: insights from a dynamic model. In: 45th IEEE Conference on Decision and Control (CDC06). pp. 2110–2115. doi:10.1109/CDC.2006.376908.

4. Lee TI, Rinaldi NJ, Robert F, Odom DT, Bar-Joseph Z, et al. (2002) Transcriptional regulatory networks in *Saccharomyces cerevisiae*. Science 298: 799–804.
